# Paediatricians knowledge, attitudes, and practices regarding immunizations for infants in Italy

**DOI:** 10.1186/1471-2458-9-463

**Published:** 2009-12-14

**Authors:** Daniela Anastasi, Gabriella Di Giuseppe, Paolo Marinelli, Italo F Angelillo

**Affiliations:** 1 Department of Public, Clinical and Preventive Medicine, Second University of Naples, Naples, (Italy)

## Abstract

**Background:**

The purpose of this study was to investigate whether paediatricians have appropriate knowledge, attitudes, and behaviours regarding vaccinations for infants in Italy.

**Methods:**

A random sample of 500 paediatricians received a self-administered anonymous questionnaire covering demographic and professional characteristics; knowledge about the mandatory, recommended, and not indicated vaccinations for infants; attitudes about vaccinations for infants; behaviour regarding current administration or willingness to administer mandatory or recommended vaccinations for infants and immunization education programs of the parents.

**Results:**

Only 42.3% paediatricians knew all recommended vaccinations for infants and this knowledge was significantly higher in females, in those who worked a higher number of hours for week, and in those who use guidelines for immunization practice. Only 10.3% had a very favourable attitude towards the utility of the recommended vaccinations for infants and this was significantly higher in those who administered recommended vaccinations for infants. A large proportion (82.7%) of paediatricians routinely informed the parents about the recommended vaccinations for infants and this appropriate behaviour was significantly higher among younger, in those with a higher number of years in practice, and in those who administered the recommended vaccinations for infants.

**Conclusion:**

Training and educational interventions are needed in order to improve knowledge, attitudes, and behaviours regarding vaccinations for infants among paediatricians.

## Background

It is very well established that the vaccinations of infants and adolescents have an extraordinary beneficial public health impact [1] by reducing morbidity, mortality, and the social and economic burden that are associated with a number of common childhood diseases [2].

In Italy, vaccinations for infants are mandatory (diphtheria, hepatitis B, polio, tetanus) and recommended (*Haemophilus influenzae *type b, measles, meningococcal, mumps, pertussis, pneumococcal meningitis, rubella, varicella) and they are administered by public health physicians and paediatricians working in a public network. All mandatory, *Haemophilus influenzae *type b, pertussis, and pneumococcal meningitis vaccines are administered according to a three-dose schedule at 3, 5, and 11-12 months of age, whereas the others with a single-dose in the second year of life. Those mandatory are actively offered free of charge whereas for the recommended the offer is independently planned by each region [3].

Health care providers are an important source of information and parents value physician recommendations also about vaccines [3]. In particular, paediatricians have a central role in the decision-making process, because they are more likely than any other health care provider to motivate and to provide appropriate educational messages to parents for their infants in order to achieve high rates of immunization [3,4]. A widespread acceptability and coverage of the vaccine in the target populations undoubtedly will also depend on paediatricians' vaccination-related knowledge, attitudes, and practices. However, very limited research has been dedicated on this topic [5-9] and it is particularly important to understand this issue in order to provide information for the development of effective immunization initiatives. Therefore, the objectives of the current study were to investigate whether paediatricians in Italy have knowledge, positive attitudes, and appropriate behaviours regarding the vaccinations for infants and to identify the variables associated.

## Methods

From October 2006 to September 2007, a cross-sectional survey was performed in a national sample of 500 paediatricians randomly recruited from the members of nine randomly selected boards of Physicians, located in the three geographical area (northern, central, southern and islands) of Italy. Study protocol and final questionnaire were approved by the ethical committee.

The questionnaire was pilot-tested on a sample of 20 paediatricians to refine the wording of items and to ensure clarity of the text. The self-administered anonymous questionnaire, revised on the basis of the comments from the pilot-study, was mailed to the random sample. Each paediatrician was approached via an introductory telephone call and was invited to participate by receiving a pack which included a letter explaining the purpose, the importance of the study, and asking them to participate, an informed consent form, a questionnaire, and a self-addressed and pre-stamped envelope for returning the questionnaire. Participants were assured that response provided would be treated in confidence. The return envelopes were coded to allow contact with non-responders, and the coding file was stored separately to preserve confidentiality and destroyed at the conclusion of the study. Non-responders received a telephone call and a further questionnaire was sent approximately two months after the initial mailing.

The questionnaire was designed to collect the following information: a) demographic and professional characteristics; b) knowledge about the mandatory, recommended or not indicated vaccinations for infants. Responses about the vaccine schedule were in an open format, whereas in the other questions respondents were asked to indicate their agreement with true or false statements; c) attitudes about vaccines were based on items measuring the perceived utility of the mandatory and recommended vaccinations for infants and beliefs regarding the severity of infectious diseases. Response to all questions on the utility of the vaccinations were on a 10-point Likert-type scale (1 = not useful at all to 10 = extremely useful), whereas the other questions were with true or false statements; d) behaviour regarding current administration or willingness to administer mandatory or recommended vaccinations and immunization education programs of the parents. Responses about current or willingness administration were in the yes/no format, whereas all other responses were measured by 5-point Likert-type scale, ranging from "never" to "always"; e) source of information about vaccination.

Participants were advised that the return of a complete questionnaire signified written consent to the study.

### Statistical analysis

Multivariable logistic regression models were constructed in order to determine significant independent predictors of the following outcomes of interest: knowledge of the recommended vaccinations for infants (Model 1); positive attitude towards the utility of recommended vaccinations for infants (Model 2); routinely inform parents regarding recommended vaccinations for infants (Model 3). In all models, the following independent variables were included: gender (male = 0, female = 1), age (continuous, in years), primary practice type (Hospital/University = 0, other = 1), number of year in practice (continuous), number of hours worked for week (continuous), number of patients seen in a workday (continuous), perception that the recommended vaccinations for infants are dangerous (no = 0, yes = 1), use of guidelines for immunization practice (no = 0, yes = 1), guidelines as source of information about vaccinations (no = 0, yes = 1), and need of additional information about vaccinations (no = 0, yes = 1). The variables knowledge of the recommended vaccinations for infants (no = 0, yes = 1) and administration of recommended vaccinations for infants (no = 0, yes = 1) were also included in Models 2 and 3.

Univariate analysis was conducted using appropriate test statistics and those characteristics associated with the outcome variables with a *p *value ≤ 0.25 were included into the multivariate analysis. The variables were added into the logistic regression models in a forward stepwise manner with inclusion criteria of *p *< 0.4 to enter the model and of *p *< 0.2 to remain in the final model. Odds ratios (ORs) and 95% confidence intervals (CIs) were calculated. Statistical significance was assessed using two-sided tests with *p*-values of ≤ 0.05. All data were analyzed using the Stata version 10 statistical software [10].

## Results

Of the 500 questionnaires, 15 were excluded because the respondent was no longer in practice and 81 because were returned due to incorrect addresses. Of the 404 eligible, 156 paediatricians completed the questionnaire for an overall response rate of 38.6%. Table 1 contains the participants' demographic and practice characteristics. The majority were females, the mean age was 52 years, more than half were in primary care, and the mean number of years in practice was 19.

**Table 1:** Selected characteristics of the study population

	n	%
Characteristic		
*Gender*		
Male	65	42
Female	91	58
*Age group, years*	51.8 ± 8 (32-79)*	
≤45	29	18.6
46-50	46	29.5
51-55	37	23.7
>55	44	28.2
*Primary practice type*		
Primary care	84	53.9
Hospital/University	54	34.6
Private practice	18	11.5
*Number of years in practice *	19.2 ± 9.4 (1-48)*	
≤10	33	21.2
11-20	64	41
21-30	42	26.9
>30	17	10.9
*Number of hours worked for week*	39.1 ± 11.7 (10-70)*	
≤30	38	24.4
31-40	69	44.2
>40	49	31.4
*Number of patients seen in a workday*	19.6 ± 9.5 (2-55)*	
≤10	37	23.7
11-15	26	16.7
16-20	32	20.5
21-25	25	16
>25	36	23.1

*Mean ± Standard deviation (range)

Table 2 summarizes the data concerning the knowledge about which vaccinations are mandatory and recommended for infants and their schedule. An adequate level of knowledge was observed for the mandatory vaccinations, with percentages ranging from 94.2% for polio and 98.7% for diphtheria; whereas lower levels were reported for the recommended with values comprised from 71.1% for varicella to 90.2% for pneumococcal meningitis. Overall, less than half (42.3%) knew all recommended vaccinations and the multiple logistic regression analysis indicated that this knowledge was significantly higher in females (OR = 2.92; 95% CI 1.36-6.27), in those working a higher number of hours for week (OR = 1.05; 95% CI 1.01-1.08), and in those who use guidelines for immunization practice (OR = 8.58; 95% CI 1.03-71.34) (Model 1 in Table 3). The responses about the schedules showed, in contrast, a lower level of knowledge. Indeed, for the mandatory vaccinations the correct number of doses were identified by 42.3% of paediatricians for polio and 78.8% for hepatitis B and for the recommended by 27.6% for meningococcal and 62.8% for varicella.

**Table 2:** Paediatricians' who know the mandatory and recommended vaccinations for infants and the immunization schedule

	Vaccination		Schedule	
	**n**	**%**	**n**	**%**

**Mandatory**				

Diphtheria	154	98.7	88	56.4
Hepatitis B	150	96.2	123	78.8
Tetanus	149	95.5	78	50
Polio	147	94.2	66	42.3
**Recommended**				
Pneumococcal meningitis	140	90.2	89	57.1
Measles	137	87.8	55	35.3
Mumps	136	87.2	55	35.3
Rubella	136	87.2	57	36.5
Meningococcal	131	84	43	27.6
*Haemophilus influenzae *type b	123	78.8	97	62.2
Pertussis	112	71.8	71	45.5
Varicella	111	71.1	98	62.8

**Table 3:** Results of the multivariate logistic regression models

Variable	OR	95% CI	***p***
Model 1. Knowledge of the recommended vaccinations for infants			
Log likelihood = -91.62, χ^2 ^= 29.31 (5 df), *p *< 0.0001			
Gender	2.92	1.36-6.27	0.006
Number of hours worked for week	1.05	1.01-1.08	0.008
Use of guidelines for immunization practice	8.58	1.03-71.34	0.047
Primary practice type	1.91	0.87-4.21	0.11
Number of patients seen in a workday	1.02	0.98-1.06	0.24
			
Model 2. Positive attitude towards the utility of recommended vaccinations for infants			
Log likelihood = -45.71, χ^2 ^= 11.76 (3 df), *p *= 0.0083			
Administration of recommended vaccinations for infants	3.31	1.11-9.85	0.031
Primary practice type	0.28	0.6-1.36	0.11
Number of patients seen in a workday	1.04	0.99-1.1	0.12
			
Model 3. Routinely inform parents regarding recommended vaccinations for infants			
Log likelihood = -59.15, χ^2 ^= 25.44 (5 df), *p *= 0.0001			
Age	0.89	0.82-0.96	0.004
Number of years in practice	1.1	1.03-1.18	0.004
Administration of recommended vaccinations for infants	6.92	1.27-37.57	0.025
Number of patients seen in a workday	1.05	0.99-1.11	0.07
Use of guidelines for immunization practice	1.91	0.48-7.61	0.36

For the perception of severity of infectious diseases, the values were, in descending order, pneumococcal meningitis (71.8%), pertussis (68.6%), diphtheria (60.9%), polio (58.3%), and hepatitis B (56.4%). The mean total values of respondents' attitude regarding the utility of mandatory vaccinations were always higher than 9, on a scale 1 to 10, indicating a very favourable attitude. In contrast, only 10.3% had a very favourable attitude by responding 9 or 10 towards the utility of those recommended and the results of the multiple logistic regression analysis indicated that this positive attitude was significantly higher in those who administered recommended vaccinations for infants (OR = 3.31; 95% CI 1.11-9.85) (Model 2 in Table 3).

With regard to the behaviours, a total of 82.7% of paediatricians self-reported that they routinely informed the parents about benefits and risks of the recommended vaccinations. In the multiple logistic regression analysis, the probability of giving such information was significantly higher in younger (OR = 0.89; 95% CI 0.82-0.96), in those with a higher number of years in practice (OR = 1.1; 95% CI 1.03-1.18), and in those who administered the recommended vaccinations for infants (OR = 6.92; 95% CI 1.27-37.57) (Model 3 in Table 3). Almost all respondents administered the mandatory vaccinations (92.9%) but only one-fourth those recommended (25.6%), whereas among those who did not administer, the mandatory were recommended by all paediatricians but the recommended were less widespread (80.8%). In particular, the lower value was observed for the varicella vaccine that was administered or recommended by only 37.2% and 34.7%. For the patients who did not receive a vaccine, 89.7% of paediatricians always ask the reason(s) for not receiving the vaccine and 78.2% recommend it again. A total of 91% and 82.7% paediatricians routinely provide information about mandatory and recommended vaccinations to their patients, respectively; whereas, a lower percentage inform about benefits and risks (77.6%).

All paediatricians reported that they have received information about vaccinations, and the main used source was scientific journals (82.7%), followed by guidelines (70.5%), and educational courses (69.2%). More than half (59.6%) said that they were interested in learning more.

## Discussion

As far as we know, this is the first observational survey specifically designed to collect detailed data on knowledge, attitudes, and behaviours regarding vaccinations for infants and to identify their determinants among a random national sample of paediatricians. The survey provides useful baseline information for further research and for policy makers.

The results of this study show that the respondents' knowledge about recommended vaccinations for infants was surprisingly low, since only 42.3% know all of them. Concern should be expressed particularly because well-informed paediatricians are essential for educating patients and for an effective health promotion of infectious diseases. Therefore, it is important that parents receive clear and consistent messages about vaccinations as a routine aspect of health care. Furthermore, a primary goal should be the development of tools in order to assist the paediatricians to provide consistent, accurate, and appropriate advice, to promote vaccinations, and to raise their knowledge about related issues. Of interest was that the multivariable analysis demonstrated that female paediatricians, those who worked a higher number of hours per week, and those who used guidelines for immunization practice were more likely to know all recommended vaccinations for infants. Some of these findings are in accordance with a previous study [9].

Surprisingly, paediatricians' attitude about the utility of recommended vaccinations for infants was not very positive because only 10.3% believed that they were extremely useful. Moreover, this positive attitude was significantly higher in those who administered these vaccinations. In this scenario, where poor recommended vaccinations related knowledge coexists with low positive attitude, one may argue that educational programs are not only needed but also welcomed. This is supported by the fact that 59.6% of respondents indicated that they desire more information about vaccinations.

Another notable finding was regarding the behaviours, with a very low proportion of the sample that routinely administered to their patients the recommended vaccinations (25.6%). Such data deserve particular attention since it can significantly increase the risk for infectious diseases. Although this data may also be partly explained by the fact that the offer of the recommended vaccinations is independently planned by each region, considering the significance of paediatrician influence and support about the health promotion of their patients, paediatricians as well as patients may play a much more powerful role in increasing infants vaccination compliance, particularly the recommended. Although 100% compliance is probably not yet a realistic goal, any increase in adherence rates will hopefully reduce morbidity and mortality in this vulnerable population. The values observed in this study were considerably lower compared with those found in paediatricians in the United States. Indeed, for children at 12 months 78% adhere to standard immunization practices [6] and 86-95% administered pneumococcal conjugate vaccine [8]. Moreover, 93% and 91% were more likely to recommend the varicella vaccine at children at age 12-18 months and 4-6 years [7] and 93% strongly recommend this vaccine routinely for children ≤ 6 years of age [11], respectively. In Switzerland, 91.6% immunized their own children with all recommended vaccines [12].

This study may have a number of methodological limitations that are worthy of emphasis and that should be considered when interpreting the results. First, the cross-sectional nature carries with it the disadvantage of not being able to prospectively identify predictors of the outcomes. Although the findings are relevant to the magnitude and direction of the relationship between the different variables and the outcomes. Rather, we can only speak of factors that are significantly associated with the outcomes. Second, the study suffers from the same problem as does any survey using results based on self-reporting instrument. Although self-report is one of the few ways to assess attitudes, there is concern about the accuracy and desirability bias with respondents that may provide responses they think the researcher wants or expects. Moreover, it is possible the tendency of the respondents for overestimation their compliance with administration/recommendation the vaccinations and, therefore, actual rates may be lower. However, we attempted to minimize these potential bias by ensuring complete respondent anonymity and confidentiality. Third, we were unable to achieve a high response rate as desired, though typical for a mailed questionnaire, even though multiple follow-up attempts were conducted, and this introduces several obvious biases that are a common limitation in mailed surveys. The information displayed by participants may be not representative of the broader population. However, no significant differences have been observed between participants and non-participants according to some demographic characteristics, so we are confident that the findings, which are based on a random sample of the population of interest, may be representative of the paediatricians in Italy.

## Conclusions

In conclusion, the findings from this study extend the understanding of the knowledge, attitudes, and behaviours regarding vaccinations for infants among paediatricians and they point out the need for designing and implementing educational programs.

## Competing interests

The authors declare that they have no competing interests.

Preliminary results were presented at the Research Meeting of the Second University of Naples, July 2-7, 2007, Naples, Italy.

## Authors' contributions

DA participated in the design of the study, collected the data, and contributed to the data analysis; GDG contributed to the data analysis and interpretation; PM participated in the design of the study and contributed to the interpretation of the data; IFA, the principal investigator, designed the study, was responsible for the data collection, statistical analysis and interpretation, and wrote the article. All authors have read and approved the final version of the manuscript.

## Pre-publication history

The pre-publication history for this paper can be accessed here:

http://www.biomedcentral.com/1471-2458/9/463/prepub

## References

[B1] Centers for Disease Control and Prevention (CDC) Impact of vaccines universally recommended for children-United States, 1990-1998 MMWR Morb Mortal Wkly Rep 1999 48 243 248 10220251

[B2] Andre FE Booy R Bock HL Clemens J Datta SK John TJ Lee BW Lolekha S Peltola H Ruff TA Santosham M Schmitt HJ Vaccination greatly reduces disease, disability, death and inequity worldwide Bull World Health Organ 2008 86 140 146 10.2471/BLT.07.040089 18297169 PMC2647387

[B3] Ministero della Salute. Calendario vaccinale http://www.ministerosalute.it/malattieInfettive/paginaInternaMenuMalattieInfettive.jsp?id=648&lingua=italiano&menu=vaccinazioni [Accessed December 22, 2008]

[B4] National Vaccine Advisory Committee Standards for child and adolescent immunization practices. National Vaccine Advisory Committee Pediatrics 2003 112 958 963 14523192

[B5] Benin AL Wisler-Scher DJ Colson E Shapiro ED Holmboe ES Qualitative analysis of mothers' decision-making about vaccines for infants: the importance of trust Pediatrics 2006 117 1532 1541 10.1542/peds.2005-1728 16651306

[B6] Koepke CP Vogel CA Kohrt AE Provider characteristics and behaviors as predictors of immunization coverage Am J Prev Med 2001 21 250 255 10.1016/S0749-3797(01)00373-7 11701293

[B7] Zimmerman RK Mieczkowski TA Mainzer HM Medsger AR Nowalk MP Understanding physician agreement with varicella immunization guidelines Prev Med 2002 35 135 142 10.1006/pmed.2002.1068 12200098

[B8] Schaffer SJ Szilagyi PG Shone LP Ambrose SJ Dunn MK Barth RD Edwards K Weinberg GA Balter S Schwartz B Physician perspectives regarding pneumococcal conjugate vaccine Pediatrics 2002 110 e68 10.1542/peds.110.6.e68 12456935

[B9] Cohen NJ Lauderdale DS Shete PB Seal JB Daum RS Physician knowledge of catch-up regimens and contraindications for childhood immunizations Pediatrics 2003 111 925 932 10.1542/peds.111.5.925 12728067

[B10] Stata Corporation. Stata Statistical Software 2007 Release 10 College Station, TX, USA

[B11] Davis MM Marin M Cowan AE Guris D Clark SJ Physician attitudes regarding breakthrough varicella disease and a potential second dose of varicella vaccine Pediatrics 2007 119 258 264 10.1542/peds.2006-0972 17272614

[B12] Posfay-Barbe KM Heininger U Aebi C Desgrandchamps D Vaudaux B Siegrist CA How do physicians immunize their own children? Differences among pediatricians and nonpediatricians Pediatrics 2005 116 e623 633 10.1542/peds.2005-0885 16263976

